# A Physiologically-Based Pharmacokinetic Simulation to Evaluate Approaches to Mitigate Efavirenz-Induced Decrease in Levonorgestrel Exposure with a Contraceptive Implant

**DOI:** 10.3390/pharmaceutics16081050

**Published:** 2024-08-07

**Authors:** Lilian W. Adeojo, Rena C. Patel, Nancy C. Sambol

**Affiliations:** 1 Department of Bioengineering and Therapeutic Sciences, School of Pharmacy, University of California San Francisco, San Francisco, CA 94143-0912, USA; lwadeojo@gmail.com; 2 Department of Medicine, University of Alabama at Birmingham, Birmingham, AL 35233, USA; renapatel@uabmc.edu

**Keywords:** simulation, PBPK model, physiologic, pharmacokinetic, levonorgestrel, efavirenz, hormone contraceptive, drug interaction, plasma protein binding

## Abstract

*Background:* Levonorgestrel implant is a highly effective hormonal contraceptive, but its efficacy may be compromised when used with cytochrome enzyme inducers such as efavirenz. The primary aim of this study was to evaluate methods of mitigating the drug interaction. *Methods:* Using a physiologically-based pharmacokinetic (PBPK) model for levonorgestrel that we developed within the Simcyp^®^ program, we evaluated a higher dose of levonorgestrel implant, a lower dose of efavirenz, and the combination of both, as possible methods to mitigate the interaction. In addition, we investigated the impact on levonorgestrel total and unbound concentrations of other events likely to be associated with efavirenz coadministration: changes in plasma protein binding of levonorgestrel (as with displacement) and high variability of efavirenz exposure (as with genetic polymorphism of its metabolism). The range of fraction unbound tested was 0.6% to 2.6%, and the range of efavirenz exposure ranged from the equivalent of 200 mg to 4800 mg doses. *Results:* Levonorgestrel plasma concentrations at any given time with a standard 150 mg implant dose are predicted to be approximately 68% of those of control when given with efavirenz 600 mg and 72% of control with efavirenz 400 mg. With double-dose levonorgestrel, the predictions are 136% and 145% of control, respectively. A decrease in levonorgestrel plasma protein binding is predicted to primarily decrease *total* levonorgestrel plasma concentrations, whereas higher efavirenz exposure is predicted to decrease *total* and *unbound* concentrations. *Conclusions:* Simulations suggest that doubling the dose of levonorgestrel, particularly in combination with 400 mg daily efavirenz, may mitigate the drug interaction. Changes in levonorgestrel plasma protein binding and efavirenz genetic polymorphism may help explain differences between model predictions and clinical data but need to be studied further.

## 1. Introduction

In 2023, an estimated 21 million females were living with human immunodeficiency virus (HIV) globally, and adolescent girls and young women accounted for more than 62% of new HIV infections in sub-Saharan Africa [1]. Additionally, 62% of their pregnancies are considered unintended and further complicated by the risk of vertical transmission of HIV [2,3]. In the last two decades, marked improvements have been made in providing more effective contraceptive methods to women in this region [4,5,6]. Subdermal implants containing either levonorgestrel or etonogestrel hormone are the most effective contraceptive methods, providing convenient birth control for three or five years with unmatched effectiveness when compared to injectables, pills, and condoms [7]. Levonorgestrel is a synthetic progesterone that is eliminated by metabolism, in part via cytochrome P450 (CYP) 3A4 (CYP3A4). Release rates from one brand of implant have been reported to be approximately 100 μg/day at 1 month, declining to 25 μg/day by year 5 [8].

In 2019, the World Health Organization recommended dolutegravir-containing antiretroviral (ARV) therapy as the preferred first-line regimen for the treatment of HIV [9]. Due to the possible association of dolutegravir with an increased risk of neural tube defect [10], its role in the treatment of women of reproductive potential needs to be factored into its risk-benefit assessment [11]. On the other hand, there are pharmacokinetic (PK) interactions between alternative ARVs and hormonal contraceptives that may render the contraceptive less effective [12,13,14,15]. Of relevance to this paper is the induction of levonorgestrel metabolism by efavirenz. Present contraception and HIV treatment guidelines advise the use of alternative or additional birth control in women taking efavirenz with a hormonal contraceptive [16].

In this in silico study, we first developed a physiologically-based pharmacokinetic (PBPK) model for levonorgestrel. PBPK models combine known quantitative measures of relevant physiologic processes (such as blood flows and abundance of specific metabolizing enzymes) with quantitative aspects of drug disposition at a cellular, tissue, or organ level (such as intrinsic liver clearance and blood to plasma partitioning), to predict PK outcomes in virtual patients given user-defined conditions. The results can be used to supplement and compare results of ongoing clinical trials, inform future trial designs, and identify gaps in our knowledge.

The primary aim of this research was to use our PBPK model for levonorgestrel, along with a pre-existing one for efavirenz, to conduct simulations that evaluate possible strategies of mitigating the interaction between efavirenz and levonorgestrel: an increase in implanted levonorgestrel dose (300 mg vs. standard 150 mg), a decrease in efavirenz oral dose (400 mg vs. 600 mg daily), or a combination of both. The lower efavirenz dose is a more recent consideration, given the results of the 630-patient, multi-nation ENCORE1 trial demonstrating that 400 mg daily has non-inferior efficacy in treating HIV disease, with fewer toxicities, than 600 mg daily [17,18,19]. The non-inferiority of the 400 mg efavirenz dose was also found in smaller trials conducted in India and China [20,21]. To date, there have not been any publications reporting the clinical drug interaction of efavirenz 400 mg daily and levonorgestrel.

New to this trial, we also evaluated the impact on levonorgestrel PK of its fraction unbound to plasma proteins as well as variations in efavirenz exposure (i.e., plasma concentrations), as might be seen with genetic polymorphisms in metabolizing enzymes.

The influence of plasma protein binding on the PK of levonorgestrel is relevant because both levonorgestrel and efavirenz are >98% bound to plasma proteins—levonorgestrel to sex hormone binding globulin (SHBG) and albumin [22] and efavirenz predominantly to albumin [23]. SHBG levels have been shown to be lower after implant placement relative to those at baseline [24,25], whereas SHBG levels increased with the use of oral levonorgestrel combined with ethinyl estradiol [26]. SHBG levels were higher when efavirenz was administered with a levonorgestrel implant [25], though the influence of HIV disease on SHBG and albumin is unknown. The current study focuses on the possibility that efavirenz competes with levonorgestrel for binding to albumin, as it does with warfarin [27], resulting in a greater fraction of levonorgestrel unbound. Plasma protein binding interactions alone usually do not translate into important clinical consequences because, for most drugs, a higher fraction unbound also results in greater clearance. Once equilibrium is re-established, there is little change to the pharmacologically active unbound concentration [28]. Recognition of protein binding alterations is, however, very important to the interpretation of drug interactions that may have a protein binding component. To better understand the role of protein binding changes on the efavirenz-levonorgestrel interaction, we evaluated the effect of levonorgestrel unbound fraction values within the range of 0.6% to 2.6% on both unbound and bound plasma concentrations of the hormone.

Because efavirenz is metabolized primarily by CYP2B6, which possesses wide genetic variations [29,30,31,32,33], and secondarily by CYP2A6 and CYP3A4 [29,34], we also explored the effects of variation in efavirenz exposure as might be seen among and within populations. The variation in efavirenz exposure was implemented by simulating both total and unbound levonorgestrel plasma concentrations with varying doses of efavirenz within the range of 200 mg to 4800 mg. 

## 2. Materials and Methods

The two main components of the study were developing a PBPK model (Section 2.1) and using the model to make predictions under alternative conditions (such as different doses) (Section 2.3). Interestingly, although levonorgestrel is an ‘old’ drug, because it was originally developed when CYP-profiling was not standard practice, in vitro data that characterize the enzymes responsible for metabolism (part of the PBPK model) were not available. A common technique to obtain missing parameter values in a PBPK model is retrograde determination—testing various values to determine which ones provide predictions of whole-body parameters (such as plasma clearance) that match those from independent reference clinical data (Section 2.2). For the reference data, rather than using one study, four informative clinical trials were pooled and analyzed to obtain whole-body plasma clearance, both with and without efavirenz.

### 2.1. PBPK Model

A PBPK model was built with Simcyp^®^ (a Certara company, Sheffield, UK; version 19.1) using the ‘minimum PKPD’ option. Physiologic parameters were selected for women ages 18 to 45 years and an average weight of 67.3 kg, reflecting a population of women of reproductive potential in sub-Saharan Africa. Most levonorgestrel-specific parameters were obtained from peer-reviewed journals and online reports (Table 1). Drug input of levonorgestrel was assumed to be zero-order and time-varying, as reported for the brand Jadelle^®^ (Bayer New Zealand, Auckland, New Zealand): 100 μg/day at one month after insertion, declining to approximately 40 μg/day within one year, 30 μg/day within three years, and 25 μg/day within five years [8].

Due to the absence of published data, levonorgestrel intrinsic clearance attributed to CYP3A4 metabolism (*CL*_int,CYP3A4_), as well as the non-CYP3A4 human liver microsomes (HLM), were determined in a retrograde fashion, by sensitivity analysis. The sensitivity analysis involved testing an array of values to find those that predicted total systemic clearance (*CL*_total_) and clearance/bioavailability (*CL*/*F*), with and without efavirenz, that best matched the reference values (next section). That is, the PBPK model parameters were optimized to predict whole-body parameters consistent with representative clinical data. For efavirenz PK and enzyme induction effects, we used model parameters that were included in the software and that had been extensively studied previously [35,36].

### 2.2. Reference PK Parameters

A mixed-effect model (MEM) analysis (NONMEM program [37], ICON plc, Ireland, version 7.4.2) was conducted on pooled mean levonorgestrel plasma concentration-time data from four trials [14,38,39,40] that reasonably represent clinical PK as well as inform, collectively, various aspects of the drug’s disposition. Mean (among subjects at each time point) concentration-time data were obtained from published graphs using GraphClick^®^ (Arizona Software, version 3.0) [41]. (Ideally, individual concentration-time data would be used, but these data were not available). The parameters obtained from this analysis, in particular *CL* and *F*, without, as well as with concomitant efavirenz, served as a reference for the development of the PBPK model.

Summaries of the study designs of the four trials and results of the MEM analysis are shown in Supplementary Tables S1 and S2, respectively. In brief, studies by Back et al., [38] Kook et al., [39] and Carten et al. [40] involved oral administration of either a single 0.25 mg dose or one or two doses of 0.75 mg levonorgestrel. The trial by Back et al. [38] also included an arm in which levonorgestrel 0.25 mg was given intravenously, thus allowing *F* to be estimated. The 2016 trial by Scarsi et al. [14] involved subdermal implants of a standard dose of 150 mg levonorgestrel with plasma concentrations collected over 48 weeks. Studies by Carten et al. and Scarsi et al. also included an arm with levonorgestrel in combination with oral efavirenz 600 mg daily. Two levels of random effects were recognized—between-study variability of *F* and within-study variability of mean plasma concentrations. The limited data regarding between-study variability did not permit estimation of inter-study variability of other parameters. The model employed a two-compartment model, with first-order absorption for oral dosing only.

### 2.3. Simulations

#### 2.3.1. Effects of Double-Dose of Levonorgestrel, Lower Dose of Efavirenz or Both

The PBPK model was used to simulate levonorgestrel plasma concentrations at 0, 1, 3, 6, 12, 24, 36, 48, and 60 months post-implant placement in 200 virtual women under each of the following conditions:

1: levonorgestrel 150 mg (75 mg × 2 rods) subdermally (control)
2: levonorgestrel 150 mg subdermally + efavirenz 600 mg orally once daily
3: levonorgestrel 150 mg subdermally + efavirenz 400 mg orally once daily
4: levonorgestrel 300 mg (75 mg × 4 rods) subdermally + efavirenz 600 mg orally once daily
5: levonorgestrel 300 mg subdermally + efavirenz 400 mg orally once daily

Efavirenz oral administration was simulated to begin at the same time as levonorgestrel administration. 

#### 2.3.2. Effects of Protein Binding and Efavirenz Exposure

We also conducted a series of simulations to test the impact of each of the following variables on levonorgestrel total and unbound plasma concentrations: (i) fraction of levonorgestrel unbound to plasma proteins and (ii) exposure of efavirenz. All simulations were done at the 1-year post-implant time point only.

Varying the fraction unbound to plasma proteins was done to evaluate the possible effects of efavirenz displacement of levonorgestrel from plasma proteins, primarily albumin. A range of unbound fractions (*f_u_*), from 0.6% to 2.6%, was evaluated because the magnitude of this interaction is unknown, as are the conditions that influence it. The lower value (0.6%) was selected as it is approximately half of the population value (1.3%); the upper bound (2.6%) was chosen as it is roughly twice the population value. Implementation within Simcyp^®^ involved setting the *f_u_* variable to each of 12 values within the range. The set of simulations was repeated for the following levonorgestrel/efavirenz doses: standard 150 mg levonorgestrel/no efavirenz, 150 mg levonorgestrel/600 mg efavirenz, and 300 mg levonorgestrel/600 mg efavirenz.

Varying efavirenz exposure (i.e., average plasma concentrations) was done to evaluate the downstream effects due to genetic differences in efavirenz metabolism. As the exact distribution of genetic variants for the most relevant enzyme, CYP2B6, differs among populations, a range of exposures was simulated. Implementation within Simcyp^®^ was done by setting the dose of efavirenz to 7 values within the range of 200 mg to 4800 mg. Another approach would have been to vary efavirenz clearance values; the results are expected to be similar because the plasma concentrations of both levonorgestrel and efavirenz fluctuate little within the day due to their long (greater than 36 h) half-lives. A 3-fold decrease in efavirenz dose (200 mg/600 mg) was chosen arbitrarily. The 8-fold increase in dose (4800 mg/600 mg) corresponds to the approximate range of steady-state trough concentrations reported in a genetic-based population pharmacokinetic study of efavirenz in HIV-1-infected individuals [33]. The set of simulations was repeated for efavirenz in combination with the standard 150 mg levonorgestrel dose as well as with 300 mg levonorgestrel. 

## 3. Results

**Figure 1:**
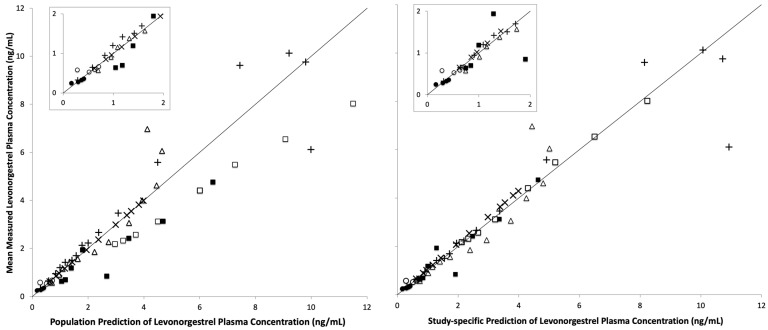
Population (**left plot**) and study-specific (**right plot**) predicted levonorgestrel plasma concentration, based on a MEM, versus observed mean concentration from one two-oral dose study without efavirenz (□) and with efavirenz (■) [40], two single-oral dose studies without efavirenz (+ and ×) [38,39], one single-IV dose study without efavirenz (∆) [38], and one implant study without efavirenz (○) and with efavirenz (●) [14]. ‘Population’ predictions (**left plot**) use point estimates of parameters for the model, whereas ‘Study-specific’ predictions (**right plot**) consider inter-study variability (i.e., use post hoc parameters). The inset plots enlarge the lower left quadrant for ease of viewing.

A MEM was used to provide reference values of *CL* and *CL*/*F* that can be compared to the *CL* and *CL*/*F* predicted with the PBPK model. The goodness of fit of the MEM model to the published mean concentration-time data is shown in Figure 1. The estimates of levonorgestrel *CL* from this model are 5.86 [95% confidence interval (CI) 4.99, 6.73] L/h without efavirenz and 10.10 (95% CI 9.11, 11.10) L/h with efavirenz. The estimates of oral *F* are 0.84 (95% CI 0.74, 0.94) without efavirenz and 0.53 (95% CI 0.43, 0.64) with efavirenz. Other parameter estimates of the MEM are shown in Supplementary Table S2. The PBPK model provided predictions of median *CL* and *CL*/*F* values comparable to those of the reference: 5.86 L/h (90% CI 5.64, 6.11) and 7.00 (90% CI 6.89, 7.53) L/h, respectively, without efavirenz, and 10.10 (90% CI 9.68, 10.44) L/h and 18.95 (90% CI 17.98, 20.00) L/h, respectively, with efavirenz.

**Figure 2:**
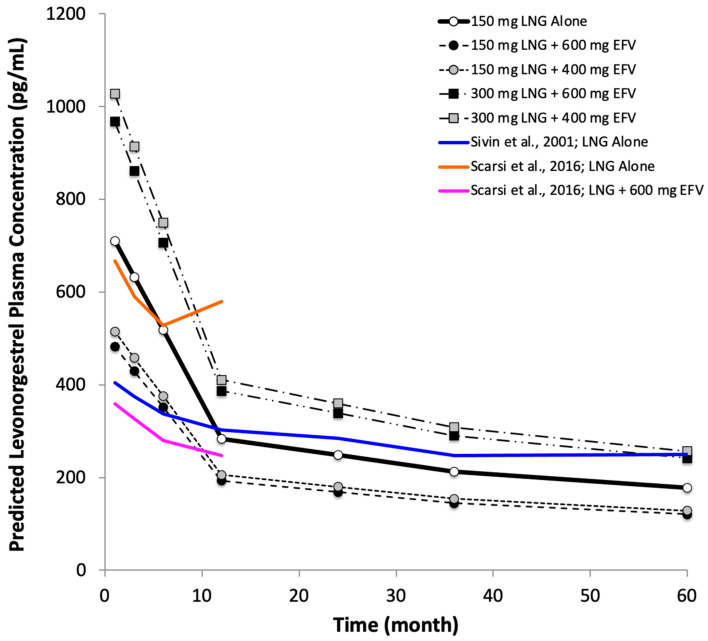
Simulated median plasma concentrations 1 month to 5 years after levonorgestrel (LNG) implant placement administered alone or with daily oral efavirenz (EFV) and superimposed mean published data for 150 mg LNG alone or with 600 mg EFV [14,42].

Levonorgestrel plasma concentrations with a 150 mg subdermal implant in the absence of efavirenz (control) are predicted to decline rapidly during the first year (Figure 2), then more gradually thereafter, as a function of the release rate. The predicted median concentration in the typical patient is approximately 284 pg/mL at 1 year, 213 pg/mL at 3 years, and 177 pg/mL at 5 years. Levonorgestrel, 150 mg with efavirenz 600 mg daily, is predicted to achieve median plasma concentrations that are 68% of control at all time points. In contrast, co-administration with efavirenz 400 mg is predicted to achieve concentrations of 72% of control. Increasing the dose of levonorgestrel to 300 mg when combined with efavirenz 600 mg daily is predicted to increase levonorgestrel plasma concentrations to approximately 136% of control, while those with efavirenz 400 mg are predicted to be 145% of control.

**Figure 3:**
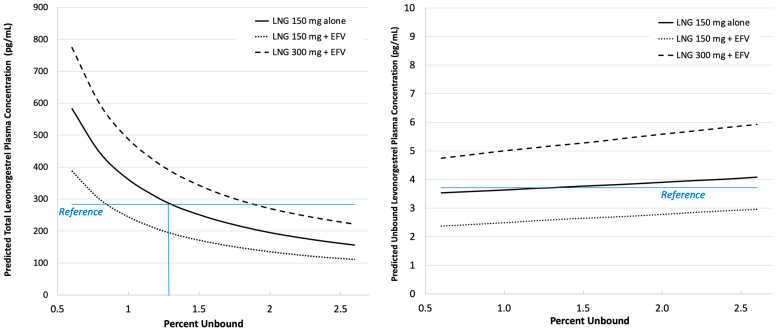
Simulated median total (**left**) and unbound (**right**) one-year levonorgestrel (LNG) plasma concentration as a function of percent unbound to plasma proteins (*x*-axis), implant dose (150 mg or 300 mg) and presence of efavirenz (EFV) 600 mg. Reference concentrations are those associated with the typical percent unbound (1.3%) for LNG 150 mg in the absence of EFV.

The 4.3-fold range of plasma levonorgestrel unbound fraction is predicted to be inversely associated with a 3.5 to 3.7-fold range of total plasma concentrations, whereas unbound plasma concentrations are predicted to vary directly and by considerably less, 1.16- to 1.25-fold (Figure 3). For the double-dose (300 mg) levonorgestrel with 600 mg efavirenz (long dashed lines), a doubling of levonorgestrel fraction unbound (i.e., *f_u_* = 2.6% vs. 1.3%) is associated with an approximately 43% lower total plasma concentration of levonorgestrel (221 pg/mL vs. 387 pg/mL) and an approximately 14% higher unbound concentration (5.92 pg/mL vs. 5.17 pg/mL). In fact, if protein binding changes are taken into account, predicted total concentrations with double-dose levonorgestrel in the presence of efavirenz may be less than standard-dose levonorgestrel without efavirenz.

**Figure 4:**
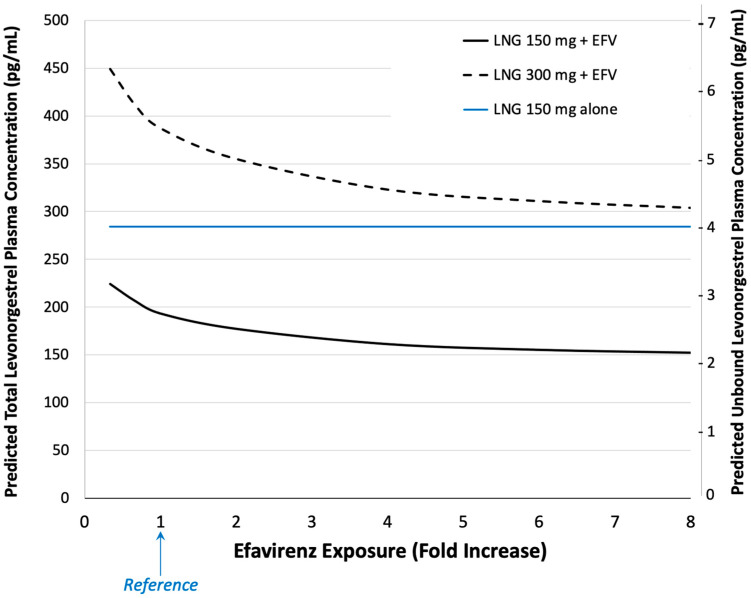
Simulated median total (left-axis) and unbound (right-axis) levonorgestrel (LNG) plasma concentration at one-year post-implant placement as a function of LNG dose and efavirenz (EFV) exposure. ‘Fold increase’ refers to varying magnitudes of EFV exposure (average plasma concentrations) associated with 600 mg for putative differing genetic subgroups relative to the general (Reference) population.

With up to an 8-fold *higher* efavirenz exposure (i.e., average plasma concentrations), as might be seen with genetic polymorphism of its *CL*, total and unbound levonorgestrel plasma concentrations are predicted to be approximately 78% of (i.e., 22% lower than) those observed with the reference exposure to efavirenz (Figure 4). For hypothetical individuals with up to 67% *lower* efavirenz exposure, levonorgestrel concentrations are predicted to be approximately 116% of (i.e., 16% higher than) those observed with the reference. The reference, in this case, refers to the typical (average) woman in the general population of mixed race/ethnicities.

## 4. Discussion 

This PBPK simulation study evaluated methods of mitigating the interaction of oral efavirenz with a levonorgestrel implant by simulating the effects of increasing the levonorgestrel dose, decreasing the efavirenz dose, and a combination of both. We also explored other variables of the interaction, namely protein binding changes of levonorgestrel and varying systemic exposure of efavirenz (reflecting genetic polymorphism of its clearance). These findings will inform future trials of levonorgestrel and efavirenz, enhance our understanding of variables that influence the drug-drug interaction, and identify gaps in our knowledge.

Of the mitigation strategies we evaluated, doubling the levonorgestrel implant dose to 300 mg is predicted to offset the interaction in the general population of women. A combination of double-dose levonorgestrel with a lower (400 mg oral daily) dose of efavirenz may be required in subpopulations having CYP2B6 variants, such as women of African descent, due to the greater induction resulting from higher efavirenz exposure. This rationale for lowering the efavirenz dose in poor metabolizers adds to the already important reason for reducing the risk of neurologic and other toxicities [43]. As noted in the Introduction, 400 mg efavirenz is non-inferior to 600 mg with respect to virological efficacy [19,20,21] and is also a recommended dose by the World Health Organization. Without an increase in the levonorgestrel dose, lowering the efavirenz dose to 400 mg oral daily alone is predicted to be inadequate for minimizing the interaction. 

A predictive model developed by Roberts et al., though somewhat different than ours, similarly projected that double-dose levonorgestrel, particularly when co-administered with efavirenz 400 mg, was effective in mitigating the drug interaction. The most notable differences in their model from ours are the upper age of the virtual population they studied (60 years vs. 45 years in our study), the efavirenz induction parameters, the limited PBPK-nature of their levonorgestrel model, reliance on one prior trial for known PK parameters (vs. four prior trials in our study), and the software used [44]. It is common for modelers to have different approaches, and both corroborating and differing results are important to establish. We also explored protein binding effects and varying exposure of efavirenz, which have not been included as part of any levonorgestrel-efavirenz interaction trial (in silico or clinical) to date.

Since the time our PBPK simulations were conducted, a clinical study of levonorgestrel-efavirenz interaction by Cirrincione et al. was published [25]. In their study, double-dose subdermal levonorgestrel in the presence of efavirenz resulted in levonorgestrel plasma concentrations that were 34% *lower* than with control (standard levonorgestrel 150 mg dose and no efavirenz). Possible explanations for the incongruities with our findings may relate to protein binding changes as well as genetic polymorphism, both of which were not included in our PBPK model but were evaluated secondarily (discussed below). Interestingly, a double-dose etonogestrel implant and efavirenz 600 mg study conducted by some of the same investigators found that the doubling strategy increased etonogestrel concentrations by 280%, and significantly fewer ovulations were detected in the double- vs. single-dose etonogestrel [45]. The contrasts between levonorgestrel and etonogestrel may be related to differences in their metabolism and genetic influences despite being similar progestins.

Levonorgestrel, delivered as an implant, provides a low-cost, convenient and effective contraception. The drug is eliminated exclusively by hepatic metabolism, with metabolites being well-characterized [46]. Though used since 1972, there are no published in vitro studies of the specific hepatic isoenzymes responsible for metabolism. Clinical interactions of levonorgestrel with known CYP3A4 inducers and inhibitors [47,48,49,50,51] provide evidence that CYP3A4 isoenzymes play a role in its metabolism. We were, however, able to estimate the contribution of CYP3A4 in a retrograde manner such that the predicted PBPK whole body parameters matched those of the reference. While the retrograde determination of missing parameter values is not new with PBPK, obtaining the references from MEM analysis of multiple clinical studies is, we believe, a novel approach [36].

In contrast, there is considerable information regarding efavirenz’s metabolism and CYP3A4 induction. Efavirenz is a first-generation non-nucleotide reverse transcriptase inhibitor that is typically combined with two other ARV drugs in a single tablet. It is primarily metabolized by CYP2B6 and secondarily by CYP2A6 and uridine 5′-diphospho-glucuronosyltransferase [34,52]. Its induction of CYP3A4 has been studied both in vitro [35,53] and clinically [54]. We used the PBPK model for efavirenz induction, which has already been incorporated into the Simcyp^®^ program [35] after being developed and systematically evaluated by Ke et al. [36]. Clinically, efavirenz also induces CYP2B6 [36,55] and, in vitro, inhibits CYP2C8 [56], but there is no evidence that levonorgestrel is metabolized appreciably by these isoenzymes. In vitro experiments and clinical sampling studies have not shown an appreciable effect of efavirenz on p-glycoprotein (P-gp) [57,58]. In contrast, a clinical interaction study with substrate probes demonstrated induction of gut enzymes, as well as slight P-gp induction [54]. Thus, we believe our efavirenz PBPK model and associated parameters to be robust.

Using the PBPK model, the predictions for total levonorgestrel plasma concentration in the *absence* of efavirenz during the period of 12 months to 5 years post-implant placement are, on average, in line with the corresponding measurements in 171 women studied by Sivin et al. [42] and, during the period 1–6 months post placement, in line with 17 women studied by Scarsi et al. [14] (Figure 2). Our predictions for levonorgestrel concentrations in the *presence* of efavirenz are higher than those observed by Scarsi [14], even though data from the Scarsi study were included in the reference MEM analysis, likely due to contributions of other data.

Our analysis suggests the portion of levonorgestrel total *CL* that is attributable to CYP3A4, in the absence of an interacting drug, is approximately 16%. Though this contribution is modest, because the induction by efavirenz is strong, with an estimate of more than a 3-fold increase in CYP3A4 activity with 600 mg oral daily doses, we predict total *CL* increases approximately 1.7-fold in a typical patient. Orally administered levonorgestrel appears even more susceptible to interaction with efavirenz, with *CL*/*F* predicted to increase approximately 2.7-fold with standard doses of efavirenz, primarily because of the presence of CYP3A4 in the gut wall.

Our basic PBPK model assumed a constant 98.7% binding of levonorgestrel to plasma proteins albumin and sex hormone binding globulin [22] due to the lack of information regarding the impact of variables such as study population (such as race/ethnicity, age, presence of HIV disease), dose level and regimen, and most importantly, the presence of efavirenz which may compete for albumin binding [23]. Thus, we explored the impact of the percent plasma protein binding of levonorgestrel on its total and unbound plasma concentrations (Figure 3). As expected [28], with increasing percent unbound, total levonorgestrel plasma concentrations are predicted to decrease, while unbound concentrations are only slightly increased. For example, with an increase of *fraction unbound* from 1.3% to 2% for levonorgestrel 150 mg (with efavirenz), predicted *total* levonorgestrel plasma concentration decreases from approximately 390 pg/mL to 285 pg/mL, whereas *unbound* concentration increases from approximately 5.2 pg/mL to 5.7 pg/mL. Efavirenz may thus have a dual effect of lowering total levonorgestrel plasma concentrations via both enzyme induction and protein binding displacement. These effects could explain why, despite doubling the levonorgestrel dose to 300 mg in the presence of 600 mg daily efavirenz in a clinical trial, total levonorgestrel concentrations were observed to be lower than those seen in the absence of an inducer/protein displacer [25].

The plasma concentration of efavirenz [29,31,32,33,34,59,60], and thus, its magnitude of induction of hormone contraceptives [61,62], may depend on the distribution of genetic polymorphisms with respect to the metabolism of CYP2B6, which is largely responsible for efavirenz elimination, and perhaps also CYP3A4 and ATP-binding cassette transporters [61]. The prevalence of at least one gene associated with slow metabolism of efavirenz (CYP2B6 516G>T polymorphism; CYP2B6*6 allele) is significantly higher in sub-Saharan Africans (30–50%) and African Americans or Europeans (12–33%) compared to Hispanics (5–7%), Caucasians (8–25%), and Asians (15%) [31,63,64,65,66,67]. These data suggest that, without a dose reduction of efavirenz, women of African descent may, on average, experience higher efavirenz exposure and subsequently lower levonorgestrel concentrations compared to their counterparts from other origins. This assertion is supported by several clinical studies [14,25,61].

To examine the impact of genetic variations in efavirenz metabolism, we conducted simulations with both lower and higher doses of efavirenz (to mimic genetic variation in metabolism) while holding other variables constant (Figure 4). The increase in levonorgestrel metabolism induction plateaus with higher efavirenz exposures due to the saturable nature of this effect and is reflected in our prediction [36,68]. With a 4-fold higher efavirenz exposure, both total and unbound levonorgestrel concentrations are predicted to be 16.6% lower than reference, whereas, with an 8-fold higher efavirenz exposure, they are 21.2% lower.

Taken together, the lower unbound levonorgestrel concentrations due to enzyme induction by efavirenz may offset the unchanged (or slightly higher) unbound concentration due to protein binding displacement, resulting in a net lower pharmacologically active, unbound levonorgestrel concentration. This hypothesis remains to be tested, as unbound levonorgestrel concentrations are not routinely measured in clinical trials. It is, however, consistent with the trend of more ovulatory activity seen with the double-dose levonorgestrel + efavirenz group compared to the standard dose levonorgestrel alone group in the Cirrincione et al. study [25]. Relative to levonorgestrel concentrations without efavirenz, a doubling of levonorgestrel in the presence of 600 mg daily efavirenz orally may not, in some individuals, sufficiently counteract the induction of levonorgestrel metabolism [25].

In closing, it should be noted that a limitation of retrograde determination of some PBPK parameters is that it is guided by clinical outcomes, and thereby diminishes predictive ability somewhat. In our case, we combined outcomes from multiple sources to minimize this limitation. As we acquire additional experimental data, we hope to strengthen the validity of those parameters and refine the model. Where clinical data were lacking or limited, as was the case with levonorgestrel plasma protein binding with concurrent efavirenz and with efavirenz genetic polymorphism, we tested a range of values to address the uncertainty. 

## 5. Conclusions

In the presence of efavirenz, doubling the dose of levonorgestrel in the general population, together with a lower dose of efavirenz in populations of African descent, holds promise of mitigating the drug-drug interaction. However, lowering the dose of efavirenz alone while maintaining the standard levonorgestrel dose is predicted to be inadequate. Further research of variables such as protein binding and genetic polymorphism of metabolism will help improve model predictions and, more importantly, therapeutic decisions. 

## Figures and Tables

**Table 1:** Values of levonorgestrel physicochemical and PK properties used in the PBPK model.

Parameter	Value	Source
B/P	0.671	calculated
*f_u_*	0.013	[22,26]
*CL*_intrinsic,CYP3A4_ (μL/min/pmol)	0.0843	RD
*f_u_*_,mic_	0.449	calculated
HLM (μL/min/mg protein)	128	RD
*Q* (L/h)	10.2	MEM
*V*_ss_ (L/kg)	2.27	MEM
Release rate ^a^ (μg/day)		
month 1	100	[8]
month 3	89 ^b^	
month 6	73 ^b^	
month 12	40	
month 24	35 ^b^	
month 36	30	
month 60	25	

Key: B/P: blood to plasma ratio; *f*_u_: fraction of unbound drug in plasma; *CL*_intrinsic,CYP3A4_: intrinsic CYP3A4 clearance; RD: retrograde determination; *f_u_*_,mic_: in vitro microsomal enzyme system fraction of drug unbound; HLM: human liver microsome elimination; *Q*: intercompartmental clearance; MEM: mixed effects model; *V*_ss_: steady-state volume of distribution; ^a^: reaching systemic circulation; ^b^: interpolated.

## Data Availability

Data are contained within the article and Supplementary Materials.

## Supplementary Materials

The following supporting information can be downloaded at: https://www.mdpi.com/article/10.3390/pharmaceutics16081050/s1, Table S1: Characteristics of Studies Used to Provide Reference Parameters; Table S2: Parameter Estimates of Mixed Effects Model Pooled Analysis used for Reference.
