# Supplementary material for: A Physiologically-Based Pharmacokinetic Simulation to Evaluate Approaches to Mitigate Efavirenz-Induced Decrease in Levonorgestrel Exposure with a Contraceptive Implant
Source: Pharmaceutics. 2024 Aug 7;16(8):1050. doi: 10.3390/pharmaceutics16081050 (PMC11359785; doi:10.3390/pharmaceutics16081050)
Supplement: Supplementary file 1 [file pharmaceutics-16-01050-s001.zip › pharmaceutics-3037974-supplementary.pdf]

pharmaceutics

Supplementary Materials for

Article

A Physiologically-Based Pharmacokinetic Simulation to Evaluate Approaches to Mitigate Efavirenz-Induced Decrease in Levonorgestrel Exposure with a Contraceptive Implant

Lilian W. Adeojo [1,†], Rena C. Patel [2] and Nancy C. Sambol [1,*]

[1] Department of Bioengineering and Therapeutic Sciences, School of Pharmacy, University of California San Francisco, San Francisco, CA 94143-0912, USA; lwadeojo@gmail.com
[2] Department of Medicine, University of Alabama at Birmingham, Birmingham, AL 35233, USA; renapatel@uabmc.edu
* Correspondence: nancy.sambol@ucsf.edu; Tel.: +01-415-476-8884
† Current Address: Arcus Biosciences, Hayward, CA 94545, USA

Supplement Table S1. Characteristics of Studies Used to Provide Reference Parameters.

Study	Reference	Number of Subjects	Dose(s) (mg)	Route	Concomitant Efavirenz	Note
Back 1987	[38]	5	0.25 mg	oral	no	in combination with ethinylestradiol
		5 (crossover)	0.25 mg	intravenous	no	in combination with ethinylestradiol
Kook 2002	[39]	16	0.75 mg	oral	no	
Carten 2012	[40]	21	0.75 mg x 2	oral	no	doses separated by 12 h; PK sampling started at 12 h
		21 (crossover)	0.75 mg x 2	oral	yes	doses separated by 12 h; PK sampling started at 12 h
Scarsi 2016	[14]	18	150 mg	subdermal	no	two rods
		20	150 mg	subdermal	yes	two rods

Supplement Table S2. Parameter Estimates of Mixed Effects Model Pooled Analysis used for Reference.

Parameter	Point Estimate	95% Confidence Interval of Point Estimate	Point Estimate Inter-Study Variability	95 % Confidence Interval Inter-Study Variability
k_a	4.15 h^{-1} *			
CL	5.86 L/h	(4.97, 6.75) L/h		
$CL_{\text{with efavirenz}}$	10.1 L/h	(9.09, 11.1) L/h		
V_{central}	49.5 L	(36.3, 62.7) L		
Q	10.2 L/h	(6.9, 13.5) L/h		
$V_{\text{peripheral}}$	105 L	(85.6, 124.4) L		
F_{oral}	0.837	0.736, 0.938	21.2 %	(0, 30.1) %
$F_{\text{oral, with efavirenz}}$	0.533	0.427, 0.639		
Intra-study variability in mean concentrations				
Additive component	0.214 ng/mL	(0.157, 0.258) ng/mL		
Proportional component	8.4 %	(0, 12.2) %		

k_a: rate constant of absorption, CL: clearance, V_{central}: volume of distribution of central compartment, Q: intercompartmental clearance, $V_{\text{peripheral}}$: volume of distribution of peripheral compartment, F_{oral}: oral bioavailability

* fixed value (not estimated) due to limited data in the absorption phase; value expected to give t_{max} of approximately 1–1.5 h, consistent with published observations
